# Cuticular Lipids as a First Barrier Defending Ixodid Ticks against Fungal Infection

**DOI:** 10.3390/jof8111177

**Published:** 2022-11-08

**Authors:** Cárita S. Ribeiro-Silva, Elen R. Muniz, Valesca H. Lima, Cíntia C. Bernardo, Walquíria Arruda, Rosane N. Castro, Patrícia S. Gôlo, Isabele C. Angelo, Éverton K. K. Fernandes

**Affiliations:** 1 Departamento de Biociências e Tecnologia, Instituto de Patologia Tropical e Saúde Pública, Universidade Federal de Goiás, Goiânia 74690-900, Brazil; 2 Faculdade Integradas da América do Sul, Caldas Novas 74692-532, Brazil; 3 Departamento de Histologia, Embriologia e Biologia Celular, Instituto de Ciências Biológicas, Universidade Federal de Goiás, Goiânia 74690-900, Brazil; 4 Departamento de Química Orgânica, Instituto de Química, Universidade Federal Rural do Rio de Janeiro, Seropédica 23890-000, Brazil; 5 Departamento de Parasitologia Animal, Instituto de Veterinária, Universidade Federal Rural do Rio de Janeiro, Seropédica 23890-000, Brazil; 6 Departamento de Epidemiologia e Saúde Pública, Instituto de Veterinária, Universidade Federal Rural do Rio de Janeiro, Seropédica 23890-000, Brazil

**Keywords:** entomopathogenic fungi, *Rhipicephalus microplus*, *Amblyomma sculptum*, *Dermacentor nitens*, *Rhipicephalus sanguineus*, lipids, hydrocarbons, biological control

## Abstract

The chemical composition of tick cuticles acts as a barrier to pathogens and may limit infection by entomopathogenic fungi. This study characterized the cuticular neutral lipids (NL) and hydrocarbons (HCs) of four ixodid ticks that are widely distributed in Brazil. HC extracts were analyzed by gas chromatography-mass spectrometry and used to challenge *Beauveria bassiana* IP361 and *Metarhizium robertsii* IP146; the effect of cuticular extracts in fungal growth were evaluated by disk diffusion and conidial viability assays. In addition, conidial germination on the tick cuticle was evaluated by scanning electron microscopy, and NL from ticks treated with fungi were assessed by thin layer chromatography. Six HCs were exclusively identified in *Amblyomma sculptum*. Additionally, cuticle extracts from *Dermacentor nitens* and *A. sculptum* inhibited the growth of *M. robertsii* IP146 and reduced conidial germination of *B. bassiana* IP361 to 70% and 49%, respectively; the same extracts also produced cytotoxic effects, with conidial death above 30% and 60%. Electron micrographs showed a delayed germination of conidia incubated for 48 h or 72 h on *D. nitens* and *A. sculptum*. The lipid profile of *A. sculptum* treated with fungi was not significantly altered; triacylglycerol was not detected in the cuticle extracts of any other tick species. Finally, *A. sculptum* and *D. nitens* cuticles have lipid components that may limit the development of *M. robertsii.*

## 1. Introduction

Ticks are distributed worldwide and can parasitize almost every class of vertebrates [1,2], and they are considered important vectors of a wide range of pathogens to animals and humans [3,4]. Ixodid tick species found in Brazil impose a high impact on public health and on the global economy: *Amblyomma sculptum* (formerly, *Amblyomma cajennense* sensu lato) (Acari, Ixodidae) transmits *Rickettsia rickettsii* (Rickettsiales: Rickettsiaceae) that causes the bacterial zoonosis known as Brazilian spotted fever [5,6], whereas *Rhipicephalus microplus* causes over three billion dollars in yearly economic losses in the cattle industry [7,8]. *Rhipicephalus sanguineus* s.l. and *Dermacentor nitens* are also vectors for many disease agents, becoming increasingly relevant to one health perspective [9,10,11].

The management of ticks and tick-borne diseases (TBDs) has been dependent on the use of chemical acaricides but the withdrawal of many pesticides due to health risks, pollution of the environment and development of resistance in tick populations has generated much interest in more sustainable, benign alternatives [9,10,11,12,13,14]. Particular attention has been given to entomopathogenic fungi, which are natural pathogens of insects, ticks and mites [12,13]. Entomopathogenic fungi have been used as mycoinsecticides against pests in the agriculture industry since 1888; in Brazil, their application exceeds three million hectares [10,15]. The efficiency of entomopathogenic fungi, such as *Metarhizium anisopliae* (Hypocreales: Clavicipitaceae) and *Beauveria bassiana* (Hypocreales: Cordycipitaceae), to control ticks has been tested under laboratory and field conditions with promising results [16,17,18,19,20]; however, the interaction between biological control agents and ticks still needs elucidation [21]. 

The variance in susceptibility of ixodid ticks to entomopathogenic fungi has been documented in the literature [22], but the underlying mechanism for this variance is poorly understood. Of the limited studies, differences in susceptibility are linked to tick gender, feeding, developmental stage and tick species [23,24]. Of particular interest is the different responses of ixodid ticks to the same entomopathogenic fungi strain such as that reported for *B. bassiana* Bb 986 (or ESALQ 986). Briefly, treated engorged females of *R. microplus* were very susceptible to *B. bassiana* Bb 986 (ca. 88% control efficacy at 10^8^ conidia mL^−1^) with a low mean larval hatch (ca. 31%) [17], whereas *A. sculptum* engorged females had a low susceptibility to this fungal strain (ca. 42% control efficacy at 10^8^ conidia mL^−1^) [25]. Treated engorged females of *D. nitens* (formerly, *Anocentor nitens*) also had a low susceptibility to Bb 986, with a high mean larval hatch (ca. 62% at 10^8^ conidia mL^−1^) [26]. Conversely, engorged females of *A. sculptum* and *R. microplus* were equally susceptible to *M. anisopliae* s.l. Ma 959 (or ESALQ 959), with a high control efficacy (>96% at 10^8^ conidia mL^−1^) [25]. Furthermore, the susceptibility to entomopathogenic fungi may also vary among different populations of the same tick species, as shown in *R. microplus* [27,28]. 

The arthropod cuticle is the first and possibly most important barrier to infection by entomopathogenic fungi [29,30] The tick cuticle is subdivided into the epicuticle and procuticle with the latter being composed of exocuticle (with several pore canals) and the endocuticle (secreted in highly organized overlaid lamellae) and is composed of an inner protein layer, an intermediate layer formed by lipids, and a wax-rich outer part produced by dermal glands [31]. These layers and their compounds act as a barrier to pathogens, and this may limit infections that influence tick survival [32,33,34,35]. On the other hand, entomopathogenic fungi such as *Metarhizium* spp. and *Beauveria* spp. have evolved specialized mechanisms for overcoming the arthropod’s defense system by the enzymatic degradation of their integument [36] and invade their hosts by direct penetration through the cuticle and natural openings [19,37,38].

In the current study, we characterized and compared the neutral lipids and hydrocarbons from the cuticle of four ixodid tick species widely distributed and most frequently found in Brazil: (1) the common human-biting tick, *A. sculptum*; (2) the cattle tick, *R. microplus*; (3) a tropical lineage of the brown dog tick, *R. sanguineus* s.l.; and (4) the tropical horse tick, *D. nitens*. We also investigated the conidial germination of *M. robertsii* and *B. bassiana* s.l. on the cuticle of engorged females of the four ixodid tick species mentioned here, and evaluated the susceptibility of fungal isolates to the total lipid compounds extracted from the tick’s cuticle.

## 2. Materials and Methods

### 2.1. Fungal Isolates, Cultivation and Preparation of Fungal Suspensions

Two fungal isolates were investigated in the current study: *M. robertsii* IP 146 *and B. bassiana* s.l. IP 361. IP code refers to fungi deposited in the Fungal Research Collection of the Laboratory of Invertebrate Pathology, at the Institute of Tropical Pathology and Public Health of the Federal University of Goiás. These fungal isolates originated from Goiás State, Center-West Brazil; IP 146 was isolated from a soil sample by using a selective medium for entomopathogenic fungi [39] and IP 361 was isolated from a live *A. sculptum* engorged female collected from a naturally infested horse [40]. Both IP 146 and IP 361 are very effective against the tick *R. microplus* [20].

The isolates were cultured in Petri plates (90 × 10 mm; Cralplast^®^, Cral Prod. for Lab. Ltda., Cotia, SP, Brazil) on potato dextrose agar medium (Difco Laboratories, Sparks, MD, USA) supplemented with 1 gL^−1^ yeast extract (Bacto™ Yeast Extract, Sparks, MD, USA) in a 12 h-light/12 h-dark cycle for 15 days at 27 ± 1 °C and with a relative humidity (RH) > 80%. Fresh conidia were harvested from the plates with a spatula and suspended in 10 mL of Tween 80^®^ 0.01% (*v*/*v*) (Sigma-Aldrich^®^, São Paulo, SP, Brazil). The conidial suspensions were vortexed for one minute, filtered through sterile cheese cloth (Cremer S.A.^©^, Blumenau, SC, Brazil), and the conidia were quantified by using a hemocytometer at 400× magnification in a Leica DM750 light microscope (Leica Microsystems, Wetzlar, Germany); the conidial concentrations were then adjusted to 1 × 10^5^ conidia mL^−1^. The viability of conidia for each suspension was assessed by the percent germination, following the methods of Braga [41] and Bernardo [19]. All tests were conducted using suspensions of conidia with a viability higher than 95%.

### 2.2. Collection of Ticks

Engorged females of *R. sanguineus* s.l. and *R. microplus* were obtained from colonies maintained on artificially infested rabbits (*Oryctolagus cuniculus*), and dairy cattle, respectively. Engorged females of *A. sculptum* and *D. nitens* were collected from naturally infested horses held in the Center for Zoonosis Control in Goiânia. The hosts were not treated with acaricides for at least 30 days before tick collections.

### 2.3. Cuticular Hydrocarbons Identification

Thirty engorged females of each tick species were individually vortexed in 0.5 mL in n-hexane for liquid chromatography (Merck^®^, Darmstadt, Germany) in conical glass tubes for 10 min, three times each group. To separate the hydrocarbons, the samples were filtered through a flash chromatography column, filled with Florisil^®^ (Sigma-Aldrich, São Paulo, SP, Brazil). GC-MS analyses were performed on a gas chromatograph coupled to a mass spectrometer (GCMS-QP 2010 Plus, Shimadzu, Kyoto, Japan). The analyses were carried out using a HP-5 capillary column of 30 mm × 0.25 mm × 0.25 µm, with helium as a carrier gas at 1.0 mL min^−1^. The GC temperature was set as: 80 °C (1 min), 5 °C min^−1^ at 290 °C, then 290 °C for 17 min. Injector, ion source and interface temperatures were 290, 250 and 310 °C, respectively. The sample injection volume was 1.0 µL, all diluted with CH2Cl2. Data acquisition and analysis were performed by using Lab Solutions Software (CS 20A) and the NIST MS search database.

### 2.4. Activity of Lipid Extracts to Mycelial Growth and Conidial Viability

The antifungal activity of lipid extracts was tested by spreading 50 μL of *M. robertsii* IP 146 or *B. bassiana* s.l. IP 361 conidial suspensions on PDA medium supplemented with chloramphenicol (500 mg L^−1^) in Petri plates (90 × 10 mm). Three 6 mm-diameter paper disks (Qualy^®^, 80/m^2^, 12.5 cm) were impregnated by immersion in 50 µL of lipids extracted from tick cuticles (the same extracts used for the GC analysis reported above). Lipid-impregnated paper disks were placed on the culture medium inoculated with fungi. Control plates received filter paper disks treated with hexane right after evaporation. Treated or control plates were incubated at 27 ± 1 °C and RH > 80% for 48 h; the fungicidal activity of a tested compound was demonstrated by the inhibition of fungal growth around the impregnated filter paper. The size of the halos was measured by using a vernier caliper (Mitutoyo, São Paulo, SP, Brazil), in its greater diameter.

The activity of lipids on the viability of conidia was also investigated. Two microliters of each conidial suspension were inoculated (without spreading) in Petri dishes containing 4 mL of PDA plus chloramphenicol. At the same inoculation point, 3 μL of the lipids extract were also inoculated; in the control group, 3 µL of hexane (solvent) were inoculated instead. The plates were incubated at 27 ± 1 °C for 24 h. A minimum of 300 conidia per plate was evaluated and counted, and the percent relative germination was determined [19,41]. Conidia were considered germinated when the germ tube was longer than the maximum conidial diameter. The experiments were conducted three times on different days.

### 2.5. Cytotoxicity of Cuticle Extracts

The cytotoxic effect of cuticle extracts to fungal conidia was assessed. Conidia of IP 146 or IP 361 were scraped from culture plates and suspended in 500 µL of tick cuticle extracts (the same used for the GC analysis reported above) and incubated overnight at room temperature (20 to 25 °C). For the control group, conidia of IP 146 or IP 361 were suspended with hexane for the same period; hexane was also used for the blank group. After exposure to the cuticle extracts or hexane (control), the conidia were resuspended in 500 µL PBS solution and propidium iodide (PI 3 µL/mL) (Sigma-Aldrich^®^, São Paulo, SP, Brazil). The viability of conidia was examined in a FACSCanto II flow cytometer (Becton Dickinson, San Jose, CA, USA) by the acquisition of 10,000 events, and the data were analyzed using Version 6.0 of the FACSDiva software.

### 2.6. Germination of Conidia on Tick Cuticle

The germination of conidia of *M. robertsii* IP 146 and *B. bassiana* IP 361 was assessed on the cuticle of *A. sculptum, D. nitens, R. microplus* or *R. sanguineus* engorged females by Scanning Electron Microscopy (SEM). Three engorged females of each tick species were fixed with adhesive tape in Petri plates and treated topically on the dorsal side by applying 50 μL of IP 146 or IP 361 conidial suspension (1.0 × 10^8^ conidia mL^−1^). Each treated engorged female was incubated at 27 ± 1 °C and RH > 90% for 24, 48 or 72 h, and processed for SEM according to Barreto [42]. Briefly, after drying with hexamethyldisilane (Electron Microscopy Sciences, Hatfield, PA, USA), the samples were placed on a stub, and coated with gold in a sputter-applicator (Denton Vacuum, Desk V); conidial germination and appressorium formation were then qualitatively assessed by using a Jeol JSM 6610 microscope, at an accelerating voltage of 5 kV.

### 2.7. Neutral Lipids from Tick Cuticle

The expression of cuticular neutral lipids from engorged females was investigated by Thin Layer Chromatography (TLC) before and after treating the tick cuticle with IP 146 or IP 361 (1.0 × 10^8^ conidia mL^−1^), as mentioned above. We also evaluated lipids present in the conidial suspensions tested. The neutral lipids on the tick cuticle or fungal suspensions were determined according to Bligh and Dyer [43].

Each group with four engorged females was vortexed in a mixture of chloroform:water (2:1, *v/v*) in glass conical centrifuge tubes (Merck KGaA^®^, Darmstadt, Germany) for one minute. Then, 0.5 mL of distilled water was added to the tubes containing the females, which were vortexed again for 1 min. A volume of 800 µL of this mixture was submitted to intermittent agitation every 5 min for 1 h; the solution was then centrifuged for 10 min at 3000 rotations per minute, and the supernatant, with the lipids, was separated from the precipitate. Two milliliters of distilled water and 1 mL of chloroform were added to the supernatants to separate the organic phase (which includes the lipids) from the aqueous phase; the solution was again centrifuged. The organic phase was separated into new tubes and dried by using N2. The neutral lipids from the entomopathogenic fungi, *M. robertsii* and *B. bassiana*, were extracted from 800 µL of conidial suspensions at 10^8^ conidia mL^−1^. The organic phase of both, tick cuticles and conidial suspensions, was resuspended in 30 μL of chloroform and applied to the silica to conduct the analysis. After running the analysis in a mixture of hexane, ethyl ether and acetic acid (60:40:1 by volume), the plate was revealed by using the Cherring compound (10% CuSO4 (*w*/*v*) and 8% H3PO4 (*v*/*v*)), and the silica was burned in a hot-air oven to mark the bands.

Densitometry was obtained by the number of pixels that the band (spot) presented using ImageMaster Total Lab software, version 1.11 (Healthcare^®^ Brazil Life Sciences, São Paulo, SP, Brazil), and the quantification of lipids per spot was determined according to Ruiz and Ochoa (1997) [44]. The quantity (µg) of lipids per engorged female was calculated by using the standard curve of each class of lipids: fatty acid (FA), cholesterol (CHO), esterified cholesterol (CHOE) and triacylglycerol (TG) (Sigma-Aldrich^®^, São Paulo, SP, Brazil), where the total amount of NL was divided by the number of females per treatment. Markers of each class of lipids investigated were applied on the plates at increasing concentrations of 2 μg, 4 μg, 6 μg, 8 μg and 10 μg. Simultaneously with fungal suspension treatment, a 10 μL aliquot of the suspension was inoculated in Petri plates (60 × 15mm) with 4 mL of PDA medium plus 0.002% (*w*/*v*) Benomyl (benzimidazole fungicide) to assess the viability of conidia [41]. Five plates were analyzed on different occasions.

### 2.8. Statistical Analysis

All data were checked for normality with the Shapiro–Wilk test by using the Easyanova package [45] in the software R (R Core Team, 2016). The differences in the diameter of halos in the disk diffusion test, as well as the difference in relative conidial germination were determined by the Kruskal–Wallis test, followed by an adapted Student’s t-test in the Easyanova package. Lipids quantities, i.e., CHOE, CHO, TG and FA detected in cuticular extracts from each tick species were also compared by the Kruskal–Wallis test, followed by an adapted Student’s t-test. The differences in mean viability of conidia from the flow cytometry analysis were evaluated by one-way analysis of variance (ANOVA), followed by Student’s *t*-test. Values of *p* less than 0.05 were considered significant.

### 2.9. Ethics Statement

The studies with *R. sanguineus* s.l. (CEUA/UFG protocol #005/2017), *R. microplus* (CEUA/UFRRJ protocol #113/2014), *A. sculptum* and *D. nitens* (CEUA/UFG protocol #055/21) were conducted in accordance with the regulations of the Ethics Committee on Animal Use (CEUA) from Universidade Federal de Goiás (UFG) and Universidade Federal Rural do Rio de Janeiro (UFRRJ). The access to Brazilian genetic heritage was approved by the Genetic Heritage Management Council (CGen) of Brazil (SisGen protocol # A420934).

## 3. Results

### 3.1. Cuticular Hydrocarbons Identification

Only hydrocarbons with more than 2% abundance (compounds with 11 to 44 carbons) of the total peaks revealed in the gas chromatography were considered for analysis in this study. The chromatogram of the compounds extracted from the cuticle of *A. sculptum* demonstrated eight peaks of hydrocarbons in which octacosyl (6.63%) and tetradecan (6.38%) had the highest percentage of area at peak. Compounds from the cuticle of *D. nitens* revealed nine peaks, among which cholesta-3,5-diene (11.32%) and pentadecane (9.32%) were the most abundant compounds. *R. microplus* demonstrated the highest percentage of eicosene (16.27%) and benzene (12.88%) among the eight peaks revealed in the chromatogram. The lowest number of peaks in the chromatogram was revealed from the cuticle of *R. sanguineus* (six), of which the compounds 1,19-eicosadiene (12.68%) and E,E,Z-1,3,12-Nonadecatriene-5,14-diol (9.2%) were the most prevalent. All hydrocarbons identified from the cuticle of the four ixodid ticks studied are listed in Table 1.

### 3.2. Activity of Lipid Extracts to Mycelial Growth and Conidial Viability

**Figure 1:**
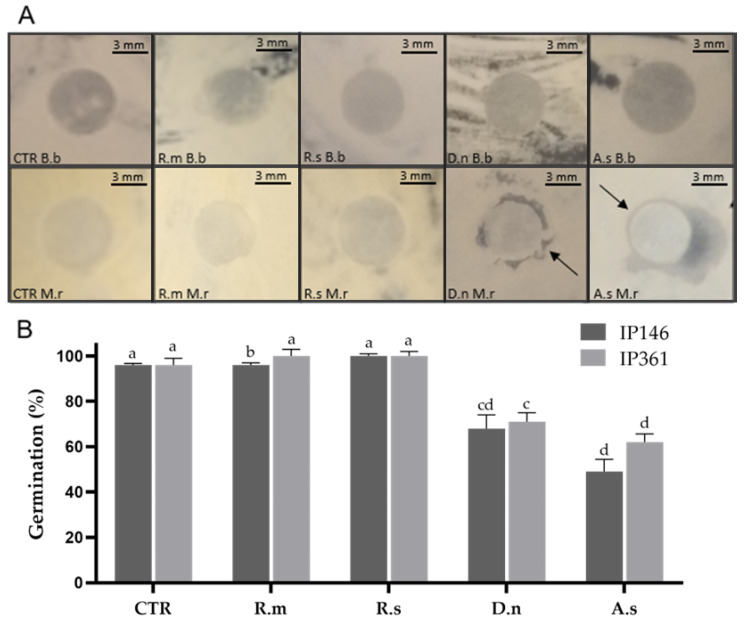
(**A**) Evaluation of the antifungal activity of the total compounds extracted from the cuticle of engorged females of *Amblyomma sculptum* (A.s), *Dermacentor nitens* (D.n), *Rhipicephalus microplus* (R.m) or *Rhipicephalus sanguineus* s.l. (R.s) at 48 h incubation. CTR Bb: *Beauveria bassiana* control; CTR Mr: *Metarhizium robertsii* control; Rm Bb: Lipid extract of *R. microplus* with *B. bassiana*; Rm Mr: Lipid extract of *R. microplus* with *M. robertsii*; Rs Bb: Lipid extract of *R. sanguineus* with *B. bassiana*; Rs Mr: Lipid extract of *R. sanguineus* with *M. robertsii*; Dn Bb: Lipid extract of *D. nitens* with *B. bassiana*; Dn Mr: Lipid extract of *D. nitens* with *M. robertsii*; As Bb: Lipid extract of *A. sculptum* with *B. bassiana*; As Mr: Lipid extract of *A. sculptum* with *M. robertsii*. Black arrows indicate inhibition of fungal growth. (**B**) Conidial germination (%) of *M. robertsii* IP 146 or *B. bassiana* IP 361 treated or not treated (control; CTR) with lipids extracted from the cuticle of engorged females at 24 h incubation. Bars with the same letter do not differ significantly (*p* > 0.05) by the Kruskal–Wallis test followed by the Student’s adjusted *t*-test.

Total extracts from the cuticle of *A. sculptum* inhibited the growth of IP 146 in disk diffusion assays; halos had a mean diameter of 4 ± 1 mm. Total extracts from *D. nitens* also inhibited the growth of IP 146 within 48 h (3 ± 1.73 mm). The growth of IP 146 was not inhibited by the extracts from the cuticle of *R. sanguineus* or *R. microplus*. The growth of *B. bassiana* was not inhibited by the extracts from the cuticle of any tick species investigated here (Figure 1).

The total extracts from the cuticle of *D. nitens* or *A. sculptum* significantly inhibited the mean conidial germination of *B. bassiana* IP 361 (71% and 62%, respectively), as well as of *M. robertsii* IP 146 (68% and 49%, respectively). Cuticular extracts from *R. microplus* or *R. sanguineus* did not inhibit the germination of either IP 146 and IP 361 after 24 h incubation, and mean germination reached exactly or approximately 100% (see Figure 1). In the control groups, mean conidial germination of fungal isolates reached virtually 100% in all replicates after 24 h incubation.

### 3.3. Cytotoxicity of Cuticle Extracts

**Figure 2:**
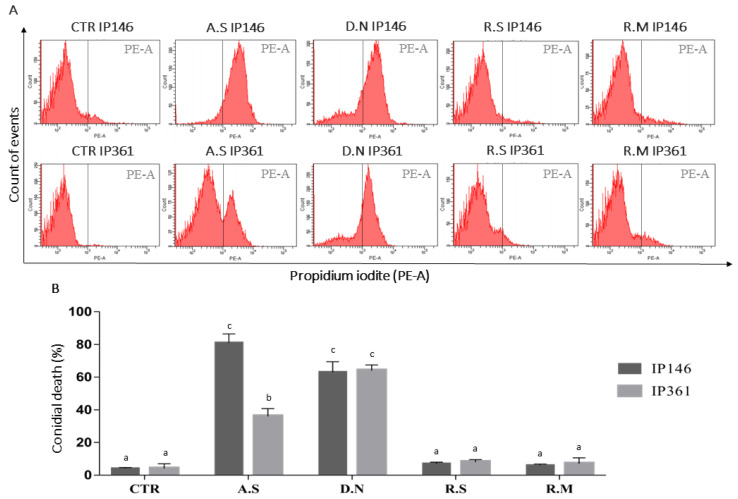
(**A**) Representative histograms showing the effects of cuticle extracts to conidia of *Metarhizium robertsii* IP 146 or *Beauveria bassiana* IP 361, treated or not treated (CTR: control) by immersion during 12 h, followed by flow cytometric analysis. (**B**) Percentage of conidial death in *M. robertsii* IP146 or *B. bassiana* IP361 after treatment with extracts from cuticle of *Amblyomma sculptum* (A.S), *Dermacentor nitens* (D.N), *Rhipicephalus microplus* (R.M) or *Rhipicephalus sanguineus* s.l. (R.S). Bars with the same letter do not differ significantly (*p* > 0.05) by analysis of variance (ANOVA) followed by the Student-Newman-Keuls (SNK) test. Each bar represents the mean of three independent experiments.

Total cuticular hydrocarbons extracted from *A. sculptum* caused significant conidial death in IP 146 (81%) and IP 361 (36%). In addition, total cuticular hydrocarbons from *D. nitens* caused conidial death in IP 146 (64%) and IP 361 (66%). Cuticular hydrocarbons extracted from *R. microplus* or *R. sanguineus*, however, caused low (<8%) conidial death in both fungal isolates, IP 146 and IP 361, which did not differ significantly from those conidia treated with hexane (control group) (Figure 2).

### 3.4. Germination of Conidia on Tick Cuticle

**Figure 3:**
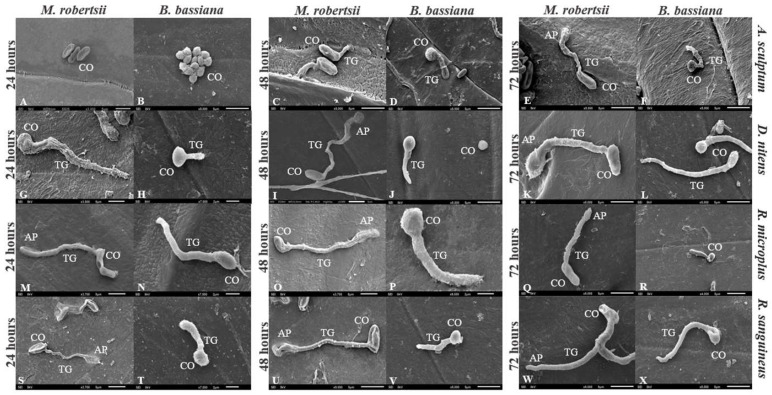
Electron micrographs of cuticles of *Amblyomma sculptum* (**A**–**F**), *Dermacentor nitens* (**G**–**L**), *Rhipicephalus microplus* (**M**–**R**) and *Rhipicephalus sanguineus* (**S**–**X**) at 24, 48 and 72 h after treatment with a conidial suspension of *Metarhizium robertsii* IP 146 or *Beauveria bassiana* IIP 361 at 5.0 × 10^8^ conidia mL^−1^. C: conidium, GT: germ tube, AP: appressorium, scale bar: 5 µm.

Conidia of both *M. robertsii* or *B. bassiana* germinated on the cuticle of treated engorged females. Conidia of IP 146 formed appressoria on the cuticle of *A. sculptum* at 72 h. On the cuticle of *D. nitens* and *R. sanguineus*, conidial germination was documented 24 h after treatment with IP 146, and appressorial formation at 48 h and 24 h, respectively, whereas IP 361 colonized the cuticle 48 h after treatment. Cuticles of *R. microplus* engorged females were fungal colonized after 24 h incubation after treatment with IP 146 or IP 361 (Figure 3).

### 3.5. Neutral Lipids from Tick Cuticle

**Figure 4:**
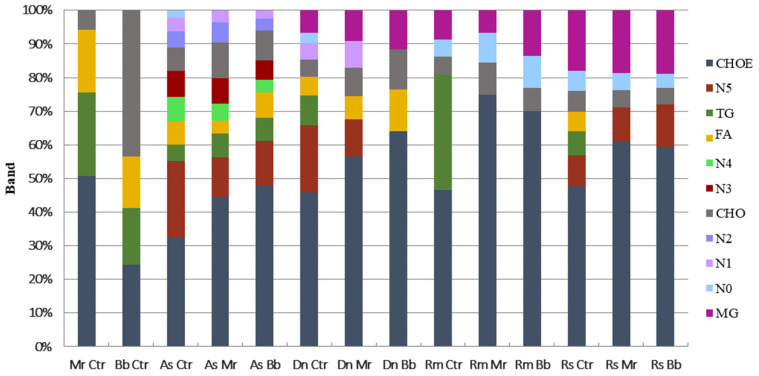
Percent of neutral lipids detected on the tick cuticle (*Amblyomma sculptum* (As), *Dermacentor nitens* (Dn), *Rhipicephalus microplus* (Rm) or *Rhipicephalus sanguineus* s.l. (Rs)) 24 h after treatment with aqueous suspensions of *Metarhizium robertsii* IP 146 (Mr), *Beauveria bassiana* IP 361 (Bb) or 0.01% Tween 80 (Ctr). Cholesterol-ester (CHOE), triacylglycerol (TG), fatty acids (FA), cholesterol (CHO), monoacyl-glycerol (MG) and unidentified (N).

Cuticular extracts from all four tick species investigated had esterified cholesterol (CHOE), triacylglycerol (TG), and cholesterol (CHO) profiles (Figure 4). Fatty acid (FA) profiles were not detected in cuticular extracts from *R. microplus* engorged females, but it was found in extracts from *A. sculptum, D. nitens* and *R. sanguineus*. Monoacylglycerol (MG) profiles were visualized in extracts from females of *R. microplus*, *R. sanguineus* and *D. nitens*. In addition, six unidentified lipid classes were detected in the extracts from *A. sculptum,* two from *R. sanguineus*, one from *R. microplus* and one from *D. nitens*. Percentage and distribution of lipid classes were calculated in consideration to the total classes within each sample. Altered lipid profiles were detected on tick cuticular extracts 24 h after treatment with IP 146 or IP 361. Accordingly, TG was not detected in extracts from treated *D. nitens, R. sanguineus* or *R. microplus*, whereas this class of lipid was found in the control groups. In extracts from *A. sculptum* (10%) and *D. nitens* (7%), FA did not differ significantly after treatment, whereas in extracts from *R. sanguineus* FA was detected only in the control groups, with approximately 3% of the total lipids extracted.

**Figure 5:**
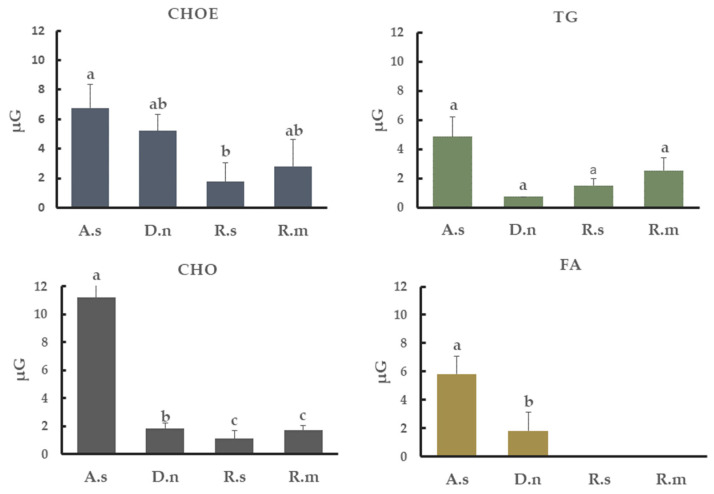
Concentration of cholesterol-ester (CHOE), triacylglycerol (TG), cholesterol (CHO) and fatty acid (FA) in the cuticle of engorged females of *Amblyomma sculptum* (A.s), *Dermacentor nitens* (D.n), *Rhipicephalus sanguineus* s.l. (R.s) or *Rhipicephalus microplus* (R.m) obtained by densitometry analysis. Bars with the same letter do not differ significantly (*p* > 0.05) by the Kruskal–Wallis test followed by the adjusted Student’s *t*-test. Each bar represents the mean of three independent experiments.

The most abundant lipid class found in cuticular extracts from all tick species studied was CHOE: 47.9% in *A. sculptum*, 58.7% in *D. nitens*, 60% in *R. microplus* and 54.8% in *R. sanguineus.* TG represented 7% in *A. sculptum*, 11.2% in *D. nitens*, 40% *in R. microplus* and 8.3% in *R. sanguineus*. The percentage of CHO found was 10% in *A. sculptum*, 6% in *D. nitens*, 7% in *R. microplus* and 8.3% *R. sanguineus*; MG was, however, 8% in *D. nitens*, 11% in *R. microplus* and 20% in *R. sanguineus*. In addition, unidentified classes of lipids extracted from the cuticle of *A. sculptum* represented more than 20% of the total extract (Figure 4). The amounts of CHOE, TG, CHO, and FA detected from each tick species were also calculated in µg per group of untreated females (Figure 5).

## 4. Discussion

The viability of fungal propagules used for arthropod pest control is crucial and may, therefore, determine the success of the infection; this process begins, in general, with the fungal germination and penetration through the host cuticle [30]. On the other hand, our findings reveal that lipid compounds on the cuticle of ixodid ticks may reduce the viability of at least some entomopathogenic fungi and limit the germination of their propagules, which may consequently limit the infection and the efficacy of biological control agents against ticks. In fact, the mean conidial germination of *M. robertsii* IP 146 and *B. bassiana* IP 361 was considerably reduced after been exposed in the laboratory to the lipid compounds extracted from the cuticle of *A. sculptum* or *D. nitens*, whereas the exposure of conidia to the cuticular lipids from *R. microplus* or *R. sanguineus* did not affect their viability. These effects might be related to the presence of lipids with fungicidal activity on the cuticle of ticks or to the ability of these fungi to use the cuticular lipids as an energy source [46,47].

The primary function of cuticular hydrocarbons is to protect arthropods against desiccation, but they also act importantly in chemical communication [48,49]. The role of hydrocarbons on the cuticular protection of ticks against invasion by pathogenic microorganisms is not well known, but this may be related to the antifungal activity of certain compounds [38,50,51]. In the present study, lipid compounds extracted directly from the cuticle of *D. nitens* or *A. sculptum* inhibited the mycelial growth of *M. robertsii* in solid medium diffusion assay. On the other hand, the total cuticular extract from *R. microplus* or *R. sanguineus* s.l. did not inhibit the growth of either IP 146 or IP 361. Squalene, a polyunsaturated hydrocarbon (C30), which corresponded to 25% of the secretory defense of the tick *Dermacentor variabilis*, did not protect it against predation by ants or caterpillars when tested individually [52]. Squalene hydrocarbon was also detected in extracts from the cuticles of *D. nitens* and *R. microplus* in the current study, but their direct effect against *M. robertsii* or *B. bassiana* was not investigated here.

On the cuticle of treated *R. microplus* and *R. sanguineus*, appressoria were detected on *M. robertsii* germ tubes 24 h after treatment. Similarly, appressoria on germinating *M. anisopliae* s.l. were observed on the *R. microplus* cuticle 24 h after treatment [53]. Evidence of penetration of *M. anisopliae* s.str. through the cuticle of *R. sanguineus* s.l., however, was not documented until 48 h after treatment [42]. In the present study, IP 146 appressoria were detected on the cuticle of *D. nitens* 48 h after treatment, whereas the formation of the appressoria of *M. robertsii* on *A. sculptum* was not seen earlier than 72 h after treatment. This longer period for detecting appressoria formation of IP 146 on *A. sculptum* (72 h) or on *D. nitens* (48 h) in comparison to *R. microplus* (24 h) or *R. sanguineus* s.l. (24 h), is possibly related to the antifungal activity of cuticular hydrocarbons on *A. sculptum* and *D. nitens* as documented by the conidial cytotoxicity (death) (see Figure 2) and by the mycelial growth inhibition of IP 146 in the disk diffusion assay (see Figure 1). This might also be the reason that geminated conidia of *B. bassiana* were not detected on the cuticle of *A. sculptum* 24 h after treatment, or may be the reason that late-germinated conidia (with a very short germ tube) were detected at 24 h on the cuticle of *D. nitens* (see Figure 2).

The ability of entomopathogenic fungi to overcome certain host antimicrobial defenses is associated with their production of enzymes that degrade the cuticle and assist fungal penetration, and to their capacity to use long-chain alkanes (between 16C and 27C) from the host cuticle as an exclusive source of carbon, which may favor the fungal infection [54,55]. In addition, cytochrome P450 monooxygenase belonging to the family CYP52 gene of *M. robertsii* was described as responsible for encoding an enzyme necessary for the efficient use of hydrocarbons from the host cuticle; once gene expression is disrupted, fungal growth is retarded. Although the *M. robertsii* genome encodes 123 cytochrome genes, only CYP52 is expressed and mediates the degradation of hydrocarbons [46].

The toxicity of cuticular extracts from *A. sculptum* or *D. nitens* to *M. robertsii* and *B. bassiana* was verified by conidial death, revealed by the binding of propidium iodide to the DNA of conidia with damaged cellular membrane. Compounds extracted from the cuticle of either *R. sanguineus* s.l. or *R. microplus*, however, were not toxic to conidia of IP 146 or IP 361. The toxicity of total hydrocarbons extracted from *A. sculptum* or *D. nitens* may be related to the activity of one compound or a combination of compounds; while many extracted hydrocarbons have been identified, their toxicities to *M. robertsii* and *B. bassiana* conidia have not been tested individually in this study. The toxicity of several cuticular hydrocarbons was tested individually in various studies: (E)-2-Hexanol and (E)-2-decanol extracted from the soybean bug, *Nezara viridula* (Hemiptera: Pentatomidae), have fungicidal activity against the conidia of *M. anisopliae* s.l. [55,56,57]. In addition, (E)-2-hexenal, (E)-2-octenal, (E)-2-decenal extracted from *Tibraca limbativentris* (Hemiptera: Pentatomidae) totally inhibited the germination of *M. anisopliae* s.l., whereas a tetradecane compound did not completely inhibit fungal mycelial growth [58]. The number of cuticular hydrocarbons on arthropod species change depending on temperature, relative humidity, food, and developmental stage, etc. [59]; it is not feasible, however, to determine a precise relationship between these variables and the diversification of hydrocarbons [48]. In addition, the mechanism of synthesis of lipid profiles is regulated by genetic factors, indicating that the cuticular compounds reveal a chemical signature for each species [60,61]. In the current study, we characterized and compared the cuticular lipid compounds of four tick species most commonly found in Brazil and neighboring countries.

The compounds produced by the four ixodid tick species investigated here are the same as those often found in insects and various other animals: CHO, CHOE, TG and FA [31]. All tick species not treated with fungi (control) had similar amounts of CHOE (ca., 55%) and TG (ca., 18%), but this class of lipids was not detected on the cuticle of engorged females of *D. nitens, R. sanguineus* and *R. microplus* that had been treated with either *B. bassiana* or *M. robertsii*. In a previous study, an insignificant amount of TG was detected in the hemolymph of infected *R. microplus*; this class of lipid, however, is regularly found in low proportions (1%) in the hemolymph of *R. microplus* [62]. In the current study, the amount of CHO differed among the tick species investigated, but not between *R. microplus* and *R. sanguineus*. Free fatty acids were not detected in *R. microplus* engorged females regardless of whether they were in the treatment or control group; conversely, fatty acids were detected on extracts from *A. sculptum* and *D. nitens*, treated or not treated with fungi, and in *R. sanguineus*, this class of lipids was detected only in the control group, which suggests that these lipids play a role in defense against pathogens. We also found six unidentified lipid bands that were unique to *A. sculptum*; conversely, monoacylglycerol profiles detected in cuticular extracts from *D. nitens*, *R. sanguineus* and *R. microplus* were not detected in *A. sculptum.* Higher amounts of FA, as well as exclusive classes of lipids, were detected in cuticular extracts from *Blatta orientalis* (Blattodea: Blattidae) than in *Blatella germanica* (Blattodea: Ectobiidae), and these results might be related to their susceptibility to fungal infection. Further, *M. anisopliae* infects cockroaches, and *B. germanica* is more susceptible to fungal infection than *B. orientalis* [63].

The current study indicates a stronger relationship between the cuticular lipid components and the susceptibility to fungi for *A. sculptum* and *D. nitens* than for *R. microplus* and *R. sanguineus* s.l., although the mechanisms in which these lipids are used in the host defense remains uncertain. Further studies will determine the damage that cuticular lipid compounds extracted from ixodid ticks cause to entomopathogenic fungi; these results may suggest improved strategies to increase the efficacy of fungal biocontrol agents against ticks and other arthropod pests. Apart from the main objective of this study, our data also raise interest in the search for components with fungicidal activity that may be explored for industrial and pharmaceutical purposes.

## 5. Conclusions

The amount of CHO on the cuticle of *A. sculptum* or *D. nitens* is higher than that found on *R. microplus* cuticle extracts. FA was only detected on *A. sculptum* and *D. nitens,* whereas MG class was detected on *R. microplus, R. sanguineus* s.l. and *D. nitens*. The total lipid compounds extracted from the cuticle of *A. sculptum* or *D. nitens* inhibited the growth of *M. robertsii*. The growth of *B. bassiana* was not inhibited when exposed to hydrocarbons extracted from the cuticle of any ixodid tick species investigated here.

## Figures and Tables

**Table 1:** Hydrocarbons identified from the cuticles of the ixodid ticks: *Amblyomma sculptum*, *Dermacentor nitens, Rhipicephalus microplus* and *Rhipicephalus sanguineus* s.l. Cuticular hydrocarbons with more than 2% abundance of the total peaks revealed by gas chromatography are listed below: (■) detected, (□) not detected.

Hydrocarbons	RT *	*A. sculptum*	*D. nitens*	*R. microplus*	*R. sanguineus*
Heneicosane	24.179	■	□	□	□
Octasiloxane	26.000	■	□	□	□
Heptadecane	28.133	■	□	■	□
Tetradecen	30.125	■	■	■	□
Tetratetracontane	28.283	■	□	□	□
Octacpsyl	28.392	■	□	□	□
Octatriacontril	29.783	■	□	□	□
Cyclononasiloxane	38.042	■	□	□	□
3,7-dimetilnonano	13.183	□	■	□	□
Pentadecane	17.758	□	■	□	□
2,3,5-trimetil Decane	17.925	□	■	□	□
Heneicosanol	26.142	□	■	□	□
9 (E)-eicoseno	22.142	□	■	□	□
1,2 Propanediol	34.067	□	■	□	□
Squalen	39.675	□	■	■	□
Cholesta 3,5 diene	40.642	□	■	■	□
Tridecan	13.000	□	□	■	□
Eicoisene	17.767	□	□	■	□
Heneicosasnol	26.142	□	□	■	□
Benzene	34.067	□	□	■	□
Pyran	39.933	□	□	■	□
9,12-Octadecadienoyl chloride	33.783	□	□	□	□
1,19 Eicosadiene	34.650	□	□	□	■
13-Octadecenal	41.417	□	□	□	■
Nonadecatriene-5,14-diol	55.273	□	□	□	■
Orixane	56.008	□	□	□	■
12-Tricosanone	56.700	□	□	□	■

(*) RT: Retention time in 59.0 min.

## Data Availability

Not applicable.

## References

[1] Parola P., Raoult D. (2001). Ticks and tickborne bacterial diseases in humans: An emerging infectious threat. Clin. Infect. Dis..

[2] Anderson J.F. (2002). The natural history of ticks. Med. Clin. N. Am..

[3] Sonenshine D.E., Roe M. (2014). Biology of Ticks.

[4] Ali A., Mulenga A., Vaz I.S. (2020). Editorial: Tick and Tick-Borne Pathogens: Molecular and Immune Targets for Control Strategies. Front. Physiol..

[5] Brito L.G., Silva Neto F.G., Oliveira M.C.S., Barbieri F.S. (2006). Bio-ecologia, Importância Médico-Veterinária e Controle de Carrapatos, Com ênfase no Carrapato dos Bovinos.

[6] Gerardi M., Ramírez-Hernández A., Binder L.C., Krawczak F.S., Gregori F., Labruna M.B. (2019). Comparative susceptibility of different populations of *Amblyomma sculptum* to *Rickettsia rickettsii*. Front. Physiol..

[7] Grisi L., Leite R.C., de Martins J.R.S., de Barros A.T.M., Andreotti R., Cançado P.H.D., Léon A.A.P., Villela H.S. (2014). Reassessment of the potential economic impact of cattle parasites in Brazil. Braz. J. Vet. Parasitol..

[8] Jongejan F., Uilenberg G. (2004). The global importance of ticks. Parasitology.

[9] De Damasceno I.A.M., Guerra R.C. (2018). *Coxiella burnetii* and Q fever in brazil: A public health issue. Ciência Saúde Coletiva.

[10] Dantas-Torres F. (2010). Biology and ecology of the brown dog tick, *Rhipicephalus sanguineus*. Parasites Vectors.

[11] Melo A.L.T., Luo T., Zhang X., Muraro L.S., Pereira N.A., Cabezas-Cruz A., Dantas-Torres F., McBride J.W., de Aguiar D.M. (2021). Serological evidence of *Ehrlichia minasensis* infection in Brazilian dogs. Acta Trop..

[12] Butt T.M., Jackson C., Magan N. (2002). Fungi as biocontrol agents: Progress, problems and potential. Educ. Psychol. Meas..

[13] Chandler D., Davidson G., Pell J.K., Ball B.V., Shaw K., Sunderland K.D. (2000). Fungal biocontrol of Acari. Biocontrol Sci. Technol..

[14] Faria M.R.D., Wraight S.P. (2007). Mycoinsecticides and Mycoacaricides: A comprehensive list with worldwide coverage and international classification of formulation types. Biol. Control.

[15] Mascarin G.M., Lopes R.B., Delalibera Í., Fernandes É.K.K., Luz C., Faria M. (2019). Current status and perspectives of fungal entomopathogens used for microbial control of arthropod pests in Brazil. J. Invertebr. Pathol..

[16] Angelo I.C., Fernandes É.K.K., Bahiense T.C., Perinotto W.M.S., Moraes A.P.R., Terra A.L.M., Bittencourt V.R.E.P. (2010). Efficiency of *Lecanicillium lecanii* to control the tick *Rhipicephalus microplus*. Vet. Parasitol..

[17] Bittencourt V.R.E.P., Peralva S.L.E.S., Viegas E.C., Alves B. (1996). Avaliaçao dos efeitos do contato de *Beauveria bassiana* (Bals.) Vuill. com ovos e larvas de *Boophilus microplus* (Canestrini, 1887) (Acari: Ixodidae). Rev. Bras. De Parasitol. Veterinária.

[18] Camargo M.G., Nogueira M.R.S., Marciano A.F., Perinotto W.M.S., Coutinho-Rodrigues C.J.B., Scott F.B., Angelo I.C., Prata M.C., Bittencourt V.R.E.P. (2016). *Metarhizium anisopliae* for controlling *Rhipicephalus microplus* ticks under field conditions. Vet. Parasitol..

[19] Bernardo C.C., Barreto L.P., Ribeiro-Silva C.S., Luz C., Arruda W., Fernandes É.K.K. (2018). Conidia and blastospores of *Metarhizium* spp. and Beauveria bassiana s.l.: Their development during the infection process and virulence against the tick Rhipicephalus Microplus. Ticks Tick-Borne Dis..

[20] Mesquita E., Marciano A.F., Corval A.R.C., Fiorotti J., Corrêa T.A., Quinelato S., Gôlo P.S. (2020). Efficacy of a native isolate of the entomopathogenic fungus *Metarhizium anisopliae* against larval tick outbreaks under semifield conditions. BioControl.

[21] Santi L., Beys da Silva W.O., Berger M., Guimarães J.A., Schrank A., Vainstein M.H. (2010). Conidial surface proteins of *Metarhizium anisopliae*: Source of activities related with toxic effects, host penetration and pathogenesis. Toxicon.

[22] Fernandes É.K.K., Bittencourt V.R.E.P. (2008). Entomopathogenic fungi against South American tick species. Exp. Appl. Acarol..

[23] Gindin G., Samish M., Zangi G., Mishoutchenko A., Glazer I. (2002). The susceptibility of different species and stages of ticks to entomopathogenic fungi. Exp. Appl. Acarol..

[24] Butt T.M., Wood M., Taylor J.W.D., Bakirci S., Hazir C., Ulug D., Hazir S. (2016). Differential susceptibility of *Hyalomma excavatum* adults and nymphs to the entomopathogens *Metarhizium anisopliae* ARSEF 4556 and *Steinernema carpocapsae*. Int. J. Pest Manag..

[25] Reis R.C.S., Melo D.R., Bittencourt V.R.E.P. (2004). Efeitos de *Beauveria bassiana* (Bals) Vuill e *Metarhizium anisopliae* (Metsc) Sorok sobre fêmeas ingurgitadas de *Amblyomma cajennense* (Fabricius, 1787) em condições de laboratório. Arq. Bras. Med. Veterinária Zootec..

[26] Monteiro S.G., Carneiro M.E., Bittencourt V.R.E.P., Daemon E. (1998). Effect of isolate 986 of the fungi *Beauveria bassiana* (Bals) Vuill on engorged females of *Anocentor nitens* Neumann, 1897 (Acari: Ixodidae). Arq. Bras. Med. Veterinária Zootec..

[27] Fernandes É.K.K., Angelo I.C., Rangel D.E.N., Bahiense T.C., Moraes Á.M.L., Roberts D.W., Bittencourt V.R.E.P. (2011). An intensive search for promising fungal biological control agents of ticks, particularly *Rhipicephalus microplus*. Vet. Parasitol..

[28] Perinotto W.M.S., Camargo M.G., Golo P.S., Angelo I.C., Quinelato S., Monteiro C.M.O., Sá F.A., Coutinho-Rodrigues C.J.B., Marciano A.F., Fiorotti J.P. (2013). Controle de *Dermacentor nitens* utilizando uma formulação comercial a base de *Metarhizium anisopliae*. Rev. Bras. Med. Vet..

[29] Butt T.M., Coates C.J., Dubovskiy I.M., Ratcliffe N.A. (2016). Entomopathogenic Fungi: New insights into host-pathogen interactions. Adv. Genet..

[30] Hartelt K., Wurst E., Collatz J., Zimmermann G., Kleespies R.G., Oehme R.M., Kimmig P., Steidle J.L.M., Mackenstedt U. (2008). Biological control of the tick *Ixodes ricinus* with entomopathogenic fungi and nematodes: Preliminary results from laboratory experiments. Int. J. Med. Microbiol..

[31] Sonenshine D.E. (1991). Biology of Ticks 1.

[32] Cherry L.M. (1969). The production of cuticle wax by engorged females of the cattle tick, *Boophilus microplus* (Canestrini). J. Exp. Biol..

[33] Hamilton J.G.C., Sonenshine D.E., Lusby W.R. (1989). Cholesteryl oleate: Mounting sex pheromone of the hard ticks *Dermacentor variabilis* (Say) (Acari: Ixodidae). J. Insect Physiol..

[34] Ment D., Gindin G., Rot A., Soroker V., Glazer I., Barel S., Samish M. (2010). Novel technique for quantifying adhesion of *Metarhizium anisopliae* conidia, to the tick cuticle. Appl. Environ. Microbiol..

[35] Ment D., Gindin G., Rot A., Eshel D., Teper-Bamnolker P., Ben-Ze’ev I., Glazer I., Samish M. (2013). Role of cuticular lipids and water-soluble compounds in tick susceptibility to *Metarhizium* infection. Biocontrol Sci. Technol..

[36] Vega F.E., Goettel M.S., Blackwell M., Chandler D., Jackson M.A., Keller S., Koike M., Maniania N.K., Monzón A., Ownley B.H. (2009). Fungal entomopathogens: New insights on their ecology. Fungal Ecol..

[37] Bittencourt V.R.E.P., Mascarenhas A.G., Faccini J.L.H. (1999). Mecanismo de infecção do fungo *Metarhizium anisopliae* no carrapato *Boophilus microplus* em condições experimentais. Ciência Rural.

[38] Kirkland B.H., Westwood G.S., Keyhani N.O. (2004). Pathogenicity of entomopathogenic fungi *Beauveria bassiana and Metarhizium anisopliae* to ixodidae tick species *Dermacentor variabilis*, *Rhipicephalus sanguineus*, and *Ixodes scapularis*. J. Med. Entomol..

[39] Rocha L.F.N., Inglis P.W., Humber R.A., Kipnis A., Luz C. (2013). Occurrence of *Metarhizium* spp. in Central Brazilian soils. J. Basic Microbiol..

[40] D’Alessandro W.B., Humber R.A., Luz C. (2012). Occurrence of pathogenic fungi to *Amblyomma cajennense* in a rural area of Central Brazil and their activities against vectors of Rocky Mountain spotted fever. Vet. Parasitol..

[41] Braga G.U.L., Flint S.D., Miller C.D., Anderson A.J., Roberts D.W. (2001). Variability in response to UV-B among species and strains of *Metarhizium* isolated from sites at latitudes from 61° N to 54° S. J. Invertebr. Pathol..

[42] Barreto L.P., Luz C., Mascarin G.M., Roberts D.W., Arruda W., Fernandes É.K.K. (2016). Effect of heat stress and oil formulation on conidial germination of *Metarhizium anisopliae* s. s. on tick cuticle and artificial medium. J. Invertebr. Pathol..

[43] Bligh G.E., Dyer W.J. (1959). A rapid method of total lipid extraction and purification. Can. J. Biochem. Physiol..

[44] Ruiz J.I., Ochoa B. (1997). Quantification in the subnanomolar range of phospholipids and neutral lipids by monodimensional thin-layer chromatography and image analysis. J. Lipid Res..

[45] Arnhold E. (2014). Package in the R environment for analysis of variance and complementary analyses. Braz. J. Vet. Res. Anim. Sci..

[46] Lin L., Fang W., Liao X., Wang F., Wei D., Leger R.J.S. (2011). The MrCYP52 cytochrome P450 monoxygenase gene of *Metarhizium robertsii* is important for utilizing insect epicuticular hydrocarbons. PLoS ONE..

[47] Keyhani N.O. (2018). Lipid biology in fungal stress and virulence: Entomopathogenic fungi. Fungal Biol..

[48] Blomquist G.J., Bagnères A.-G. (2010). Insect Hydrocarbons: Biology, Biochemistry, and Chemical Ecology.

[49] Gibbs A.G. (2002). Lipid melting and cuticular permeability: New insights into an old problem. J. Insect Physiol..

[50] Ment D., Gindin G., Soroker V., Glazer I., Rot A., Samish M. (2010). *Metarhizium anisopliae* conidial responses to lipids from tick cuticle and tick mammalian host surface. J. Invertebr. Pathol..

[51] Ment D., Churchill A.C.L., Gindin G., Belausov E., Glazer I., Rehner S.A., Rot A., Donzelli B.G.G., Samish M. (2012). Resistant ticks inhibit *Metarhizium* infection prior to haemocoel invasion by reducing fungal viability on the cuticle surface. Environ. Microbiol..

[52] Yoder J.A., Domingus J.L. (2003). Identification of hydrocarbons that protect ticks (Acari: Ixodidae) against fire ants (Hymenoptera: Formicidae), but not lizards (Squamata: Polychrotidae), in an allomonal defense secretion. Int. J. Acarol..

[53] Arruda W., Lübeck I., Schrank A., Vainstein M.H. (2005). Morphological alterations of *Metarhizium anisopliae* during penetration of *Boophilus microplus* ticks. Exp. Appl. Acarol..

[54] Pedrini N., Zhang S., Juárez M.P., Keyhani N.O. (2010). Molecular characterization and expression analysis of a suite of cytochrome P450 enzymes implicated in insect hydrocarbon degradation in the entomopathogenic fungus *Beauveria bassiana*. Microbiology.

[55] Leger R.J.S., Cooper R.M., Charnley A.K. (1988). Utilization of alkanes by entomopathogenic fungi. J. Invertebr. Pathol..

[56] Sosa-Gomez D.R., Boucias D.G., Nation J.L. (1997). Attachment of *Metarhizium anisopliae* to the southern green stink bug *Nezara viridula* cuticle and fungistatic effect of cuticular lipids and aldehydes. J. Invertebr. Pathol..

[57] Borges M., Aldrich J.R. (1992). Instar-specific defensive secretions of stink bugs (Heteroptera: Pentatomidae). Experientia.

[58] Silva R.A., Quintela E.D., Mascarin G.M., Pedrini N., Lião L.M., Ferri P.H. (2015). Unveiling chemical defense in the rice stalk stink bug against the entomopathogenic fungus *Metarhizium anisopliae*. J. Invertebr. Pathol..

[59] Dietemann V., Peeters C., Liebig J., Thivet V., Hölldobler B. (2003). Cuticular hydrocarbons mediate discrimination of reproductives and nonreproductives in the ant *Myrmecia Gulosa*. Proc. Natl. Acad. Sci. USA.

[60] Estrada-Peña A., Guglielmone A.A., Mangold A.J., Castellá J. (1993). Patterns of cuticular hydrocarbon variation and genetic similarity between natural populations of *Amblyomma cajennense* (Acari: Ixodidae). Acta Trop..

[61] Estrada-Peña A., Estrada-Peña R., Peiró J.M. (1992). Differentiation of Rhipicephalus ticks (Acari: Ixodidae) by gas chromatography of cuticular hydrocarbons. J. Parasitol..

[62] Maya-Monteiro C.M., Daffre S., Logullo C., Lara F.A., Alves E.W., Capurro M.L., Zingali R., Almeida I.C., Oliveira P.L. (2000). HeLp, a Heme Lipoprotein from the Hemolymph of the Cattle Tick, *Boophilus microplus*. J. Biol. Chem..

[63] Gutierrez A.C., Gołębiowski M., Pennisi M., Peterson G., García J.J., Manfrino R.G., López Lastra C.C. (2015). Cuticle fatty acid composition and differential susceptibility of three species of cockroaches to the entomopathogenic fungi *Metarhizium anisopliae* (Ascomycota, Hypocreales). J. Econ. Entomol..

